# Using single‐cell genomics to understand developmental processes and cell fate decisions

**DOI:** 10.15252/msb.20178046

**Published:** 2018-04-16

**Authors:** Jonathan A Griffiths, Antonio Scialdone, John C Marioni

**Affiliations:** ^1^ Cancer Research UK Cambridge Institute University of Cambridge Cambridge UK; ^2^ EMBL‐European Bioinformatics Institute (EMBL‐EBI) Wellcome Genome Campus Hinxton UK; ^3^ Institute of Epigenetics and Stem Cells Helmholtz Zentrum München München Germany; ^4^ Institute of Functional Epigenetics Helmholtz Zentrum München München Germany; ^5^ Institute of Computational Biology Helmholtz Zentrum München München Germany; ^6^ Wellcome Trust Sanger Institute Wellcome Genome Campus Hinxton UK

**Keywords:** cell fate, development, differentiation, single‐cell RNA‐seq, transcriptome, Chromatin, Epigenetics, Genomics & Functional Genomics, Development & Differentiation, Genome-Scale & Integrative Biology

## Abstract

High‐throughput *‐omics* techniques have revolutionised biology, allowing for thorough and unbiased characterisation of the molecular states of biological systems. However, cellular decision‐making is inherently a unicellular process to which “bulk” ‐omics techniques are poorly suited, as they capture ensemble averages of cell states. Recently developed single‐cell methods bridge this gap, allowing high‐throughput molecular surveys of individual cells. In this review, we cover core concepts of analysis of single‐cell gene expression data and highlight areas of developmental biology where single‐cell techniques have made important contributions. These include understanding of cell‐to‐cell heterogeneity, the tracing of differentiation pathways, quantification of gene expression from specific alleles, and the future directions of cell lineage tracing and spatial gene expression analysis.

## Introduction

High‐throughput *‐omics* techniques have revolutionised molecular biology, providing insight at every step of the central dogma. At the level of DNA, we now know the genome sequences for many species and how these vary between individuals of these species (The 1000 Genomes Project Consortium, 2015). Differences in gene expression between organisms, tissues and disease states have been extensively quantified by microarrays and RNA‐seq (for both coding and non‐coding transcripts), while mass spectrometry and other approaches have begun to yield a high‐throughput overview of protein expression. Other techniques reveal how each level of the dogma affects the other: where protein binds DNA (Aparicio *et al*, 2004; Johnson *et al*, 2007), how DNA conformation affects gene expression (Belton *et al*, 2012) and which RNA molecules are being translated (Ingolia *et al*, 2009).

However, these approaches typically require as input hundreds to millions of cells, revealing only an average reading across cell populations. For developmental biology, where individual cells make decisions about their fate, these ensemble measures provide only limited information, as individual cellular measurements are lost. Nonetheless, procedures such as fluorescence‐activated cell sorting enable isolation of specifically labelled cell populations. Isolation of specific cell types or subpopulations allows for meaningful bulk genomic analysis and has contributed a great deal to our understanding of developmental biology (Spitz & Furlong, 2006), albeit large numbers of input cells are required.

Recently developed single‐cell *‐omics* techniques (Tang *et al*, 2009; Smallwood *et al*, 2014; Buenrostro *et al*, 2015b; Heath *et al*, 2016), by contrast, are particularly apposite for developmental biology, transferring high‐throughput molecular techniques onto the correct scale for understanding cellular decision‐making. In particular, knowledge of the set of genes that different cells express allows characterisation of cell state, thus providing a direct read‐out of how dynamic decisions are made. Transcriptional information can be supplemented with the results of other assays, such as chromatin accessibility (Buenrostro *et al*, 2015a), allowing even deeper insight into the mechanisms by which cell fate is regulated.

This review focusses on transcriptomic assays, which make up the large majority of single‐cell genomic research published to date. We first summarise the processes involved in generating and analysing single‐cell expression data. We then identify areas of developmental biology where these assays have provided unique insight, as well as outlining future challenges and opportunities.

## Generating single‐cell transcriptomic data

Quantifying gene expression via microscopy is familiar in contemporary biology, whether using hybridisation techniques or artificially created fusion proteins. Flow cytometry scales up optical approaches to hundreds of thousands of cell measurements without compromising cellular resolution (Fulwyler, 1965). Historically, these methods have not been suitable for assaying many genes simultaneously, due to constraints imposed by fluorophore emission spectra. Nucleotide‐focussed methods pushed beyond this limitation: real‐time PCR (Van Gelder *et al*, 1990) can quantify hundreds of genes, with cellular throughput improved using microfluidic systems (White *et al*, 2011; Sanchez‐Freire *et al*, 2012). The recent development of sequencing‐by‐hybridisation (described later in this review) has addressed the gene‐throughput problems of optical approaches, allowing the quantification of thousands of transcripts in the same cell.

**Figure 1:**
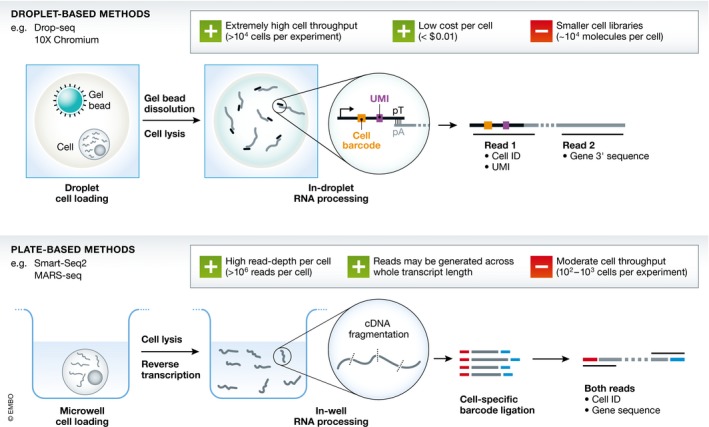
Single‐cell library preparation summary There are two primary methods for generating single‐cell transcriptomics data: plate‐based and droplet‐based methods, shown above. In summary, droplet‐based approaches offer high cell throughput, while plate‐based approaches provide higher resolution in each individual cell. Note that different implementations of these methods provide slightly different outputs and that some steps are excluded for clarity (e.g. cDNA amplification).

To achieve truly transcriptome‐wide expression coverage, however, RNA‐sequencing‐based methods are best suited. Shortly after the first application of RNA‐seq to bulk populations of cells (Bainbridge *et al*, 2006), the feasibility of applying RNA‐seq to individual cells was demonstrated (Tang *et al*, 2009). Over the past 5 years, single‐cell RNA‐seq (scRNA‐seq) has become the most commonly used approach for assaying single‐cell gene expression profiles. There are two broad sets of methods for applying single‐cell RNA‐seq—“plate‐based” and “droplet‐based” (Fig 1).

Initially, most studies used plate‐based assays, where library preparation is performed manually on cells sorted into and lysed in individual wells of a microwell plate (Jaitin *et al*, 2014; Picelli *et al*, 2014). Robotic and microfluidic systems (e.g. Fluidigm C1) have been developed to automate some of these processes.

Droplet‐based methods employ microfluidics to capture individual cells in nanolitre‐sized droplets, each loaded with reagents and unique labels: reverse transcription and transcript labelling take place within these small volumes. The droplet suspension is later broken down for pooling of cell libraries prior to sequencing. These methods have been developed by academic groups (Klein *et al*, 2015; Macosko *et al*, 2015) and commercially, by 10X Genomics (Zheng *et al*, 2017).

Each approach has its own advantages and disadvantages. Plate‐based methods tend to provide higher‐quality libraries at the cost of lower cellular throughput, processing hundreds or thousands of cells compared to the hundreds of thousands that droplet methods can process. More subtle differences also differentiate the two sets of methods. To capture rare cell types with known cell‐surface markers, it is generally more efficient to flow‐sort and prepare plates of single‐cell libraries rather than to capture more cells using a droplet method. Additionally, current droplet methods capture gene information exclusively from the 3′ or 5′ end of each transcript, while plate approaches can generate reads from across entire transcripts; the latter allows splice‐variant and allele‐specific transcriptional information to be retrieved. Finally, droplet methods are more likely to produce “multiplet” cell transcriptomes, where multiple different cells become labelled with the same barcode. This is largely due to the lack of user oversight (e.g. it is more difficult to identify attached pairs of cells) and the possible reuse of cell barcodes from the labelling beads. The doublet rate in droplet experiments is proportional to the number of loaded cells (Zheng *et al*, 2017).

For a researcher, the decision about which method to use is typically driven by the nature of the biological system under consideration—whether the quality or quantity of cells is important. For example, plate‐based methods may be more suitable for young embryos, given the small number of cells present. For later stages of development, where there are tens of thousands of cells and a higher level of heterogeneity in each embryo, a droplet method is better suited because it is relatively easy to capture a greater number of cells, which facilitates a more complete sampling and allows unbiased capture of rare cell types. Additionally, droplet methods may be preferable for studying continuous systems, as the higher number of cells sampled can be used to better approximate the continuous process that is being studied.

Both methods exploit cell‐specific DNA barcodes to allow the pooling of libraries from different cells prior to sequencing. These barcodes allow different transcriptomic reads to be assigned to individual cells. Both can also exploit unique molecular identifiers (UMIs): small, randomly generated nucleotide sequences that allow PCR duplicate reads to be collapsed, providing a more precise estimate of the actual number of RNA molecules present in a sample. For an in‐depth discussion of existing approaches, see Svensson *et al* (2017).

A new method of library preparation holds much promise for combining the benefits of both plate and droplet approaches. Here, pools of cells are repeatedly split and randomly allocated to different sets of barcodes, combinatorially building up a large diversity of possible barcode labels. The method's utility has been demonstrated for DNA sequencing (Vitak *et al*, 2017), RNA‐seq (Cao *et al*, 2017) and chromatin accessability assays (Cusanovich *et al*, 2015).

### Multi‐omic assays

The vast majority of single‐cell genomics research has focussed on capturing only RNA. However, several protocols exist that allow integration of genomic, epigenomic and transcriptional information from the same cells. For example, G&T‐seq (Macaulay *et al*, 2015) combines DNA sequencing with RNA‐seq and is adept at identifying how copy‐number changes may impact transcription. M&T‐seq (Angermueller *et al*, 2016) captures DNA methylation and transcriptome data, with NMT‐seq (preprint: Clark *et al*, 2018) further adding chromatin‐accessibility information using a GpC methyltransferase (Kelly *et al*, 2012). While these assays offer unique advantages, they are typically experimentally challenging to run, and handle many fewer cells than scRNA‐seq.

## State‐of‐the‐art analysis techniques

### Quality control

After demultiplexing barcodes and alignment of suitably trimmed reads to the appropriate reference genome, the resulting data from an scRNA‐seq experiment can be represented as an integer matrix of gene expression levels, with each entry representing the number of sequenced reads (or molecules, if UMIs were used) assigned to a particular gene in a specific cell. Notably, barcode decomposition is not trivial—particularly for the random sequences of UMIs—as sequencing errors can alter their observed sequences. Methods have been developed to account for this by predicting which barcodes have arisen by error and which truly existed within the sample (Smith *et al*, 2017).

Subsequently, it is important to assess the quality of the transcriptome for each cell: incomplete cell lysis or failures during library preparation can provide output that confounds analyses. There are many parameters that quality control (QC) tests may focus on, but there are three attributes that may be easily assessed in all single‐cell data sets: the total number of transcripts detected; the total number of genes found to be expressed; and the fraction of expression contributed by mitochondrial genes. Cells that show aberrant behaviour for these characteristics are typically removed from further analysis, albeit care must be taken when studying a heterogeneous population of cells as total mRNA content and other features can vary substantially (Ilicic *et al*, 2016).

Drop‐out is a phenomenon observed in scRNA‐seq whereby cells that are expected to express a certain gene show an observed count of zero. This is most commonly understood to be driven by stochastic failures of transcripts to be reverse‐transcribed or amplified, and therefore never sequenced. This is of particular importance for data generated by droplet assays, where capture efficiency varies considerably across cells. In order to recover expression values from dropped‐out genes, it is possible to impute expression values from other cells that show similar expression patterns (preprint: Dijk *et al*, 2017). However, the user should make sure that weak signals are not being artificially inflated. A researcher must also be aware of the possibility that doublets can drive technical signal in a data set, particularly for droplet‐based methods. While there are no published methods for doublet detection at the time of writing, a number of papers have implemented heuristic approaches for excluding multiplet libraries. These include rejecting cells expressing sets of biologically mutually exclusive markers (e.g. *Xist* and Y chromosome genes; Ibarra‐Soria *et al*, 2017), and by identifying small clusters composed of cells with large library size whose expression profiles correlate strongly with at least two other clusters in the data set (Bach *et al*, 2017).

### Confounding factors

Single‐cell RNA‐seq experiments are sensitive to confounding factors. For example, as in any ‐omics experiment, systematic differences between experimental batches must be removed before the expression profiles of cells can be compared, emphasising the importance of good experimental design (Lun & Marioni, 2017). Even when controlling for these effects, true biological differences may produce signals orthogonal to the experiment's aim. In particular, cell size (as reflected by total mRNA content) often manifests itself in the number of detected genes in each cell (McDavid *et al*, 2016; Hicks *et al*, 2017), which can lead to structure in the high‐dimensional expression space. Cell library size differences are controlled by the critical step of normalisation (reviewed in Vallejos *et al* (2017)), which aims to remove differences due to sequencing depth and total RNA content. The addition of precisely quantified exogenous RNA species (“spike‐in” genes) to each cell's lysate allows the estimation of absolute amounts of RNA (Brennecke *et al*, 2013). However, their use is rare in droplet‐based assays: spike‐in RNA will be present in every droplet, not only those containing cells. Consequentially, spike‐in genes may consume a large amount of the sequencing read space and would be confounded by repeated use of the same cell barcode in multiple droplets (resulting in a variable amount of spike per barcode). Other biological factors such as cell‐cycle stage can also lead to structure that can mask the signal of interest; computational strategies exist to identify and remove these effects (Buettner *et al*, 2015).

### Cell type identification

A common first step in the analysis of scRNA‐seq data is to classify cells into a number of groups. By identifying these subgroups of cells, the degree of heterogeneity within the population of interest can be assessed and comparisons can be performed, even between potentially small or rare groups of cells (e.g. primordial germ cells).

Cell‐type clustering performance can be improved by using only genes that vary more between cells than would be expected by chance (Brennecke *et al*, 2013), or by using “eigengenes” that explain variability in the data (e.g. derived via principal components analysis). For additional discussion of these features, see Trapnell (2015).

### Developmental trajectories and pseudotime

In many systems, cells display a continuous spectrum of states that is considered to represent the differentiation process. In these cases, a discrete classification of cells is not appropriate, and a researcher may prefer to use a method that summarises the continuity of cell states in the data.

**Figure 2:**
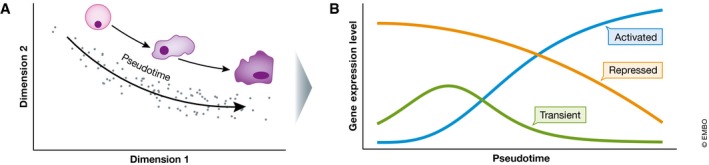
Pseudotime recapitulates developmental trajectories (A) By observing similarities between the expression profiles of cells, it is possible to order cells along an axis of pseudotime that recapitulates developmental processes. (B) Having established this ordering, genes that show significant changes in expression along the developmental pathway may be identified.

Such methods are typically referred to as *pseudotime* methods, a term first introduced by the software package Monocle (Trapnell *et al*, 2014). Pseudotime describes an ordering of cells according to some characteristic in the data; this may represent developmental processes occurring over time, or the effects of continuous spatial heterogeneity in a system. Because pseudotime is an ordering of cells, it allows identification of the cell types at the beginning and end states of the trajectory, as well as those cells in intermediate stages (Fig 2). From the ordering of cells, it is possible to identify the transcriptional changes that accompany developmental processes, which can also permit the reconstruction of gene regulatory networks (Moignard *et al*, 2015). Additionally, recent developments allow detection of branching points in trajectories (Haghverdi *et al*, 2016), which serve to identify critical points of cellular decision‐making. Note that caution must be exercised when applying classification and pseudotime methods, as they are guaranteed to generate output irrespective of the quality of data supplied. There is rarely any quantification of uncertainty, and results typically depend on specific parameter choices. For proper interpretation, it is important to ensure that input data are of high quality and not confounded by, for example, batch effects. Moreover, it should be stressed that scRNA‐seq's static “snapshot” data possess intrinsic limitations for the study of dynamic processes, which are common throughout developmental biology (preprint: Weinreb *et al*, 2017).

## The contribution of single‐cell expression data to developmental biology

In this section, we highlight examples from developmental biology where the application of single‐cell gene expression assays has played a key role in providing new biological insights.

### Understanding cellular heterogeneity

There are two ways to look at scRNA‐seq data: how the expression profiles of individual cells differ from each other, and what structure in the data drives this; or how different genes behave across the population of cells and with respect to other genes’ expression. In this section, we describe how cultured mouse embryonic stem cells have been used as a model for understanding the role of dynamic gene expression patterns, before discussing how expression variability observed between cells in mouse embryos defines cell fate choices in early development.

#### Observing heterogeneity in cultured cells

Embryonic stem cells are a foundational tool of developmental biology research, offering a platform to investigate specific cell fate choices by signal‐induced differentiation. Early work on mouse embryonic stem cells (mESCs) identified archetypal gene expression patterns across cells, highlighting bimodal and lognormally expressed genes (which were typically pluripotency regulators) as well as sporadically expressed transcripts (mostly differentiation markers; Kumar *et al*, 2014). It is difficult to address the dynamics of cellular gene expression from scRNA‐seq data alone, as it captures only snapshots of cells’ gene expression (preprint: Weinreb *et al*, 2017). To address this, Kumar *et al* allowed individual cells to grow into colonies over 3 days and quantified the expression levels of key pluripotency genes in individual cells of each colony. A higher level of inter‐colony variance than intra‐colony variance was observed, demonstrating that the initial gene expression differences that existed within the originating cells had not been overcome by gene expression pattern changes over the course of several cell cycles. The rate of change of pluripotency markers was therefore shown to be relatively slow.

Further work in mESCs focussed on identifying differences between cell culture conditions: a foetal calf serum + LIF environment promotes self‐renewal in stem cells, while adding additional inhibitors (“2i”) further prevents differentiation. Cells treated in each of these conditions were profiled using scRNA‐seq (Kołodziejczyk *et al*, 2015). Although global levels of gene expression variability were equivalent between environments, specific functional groups of genes were more or less variable in each condition. Gene ontology terms such as “organ development” were more variably expressed in the serum condition, where differentiation is less repressed, while 2i‐treated cells showed greater variability in the expression of cell‐cycle genes. Whole‐transcriptome comparisons additionally revealed that the different treatments produce distinct transcriptome profiles, suggesting no overlap between subpopulations of serum‐treated and 2i‐treated cells, as was previously thought to be the case.

#### Heterogeneity *in vivo*

To form the axes that define embryonic structure, an embryo must break the initial symmetry of the zygote. The degree to which stochastic fluctuations in gene expression bias cell fate in symmetry breaking is controversial (Hadjantonakis & Arias, 2016), so application of single‐cell approaches is particularly appropriate.

An analysis of mouse embryonic cells (from the zygote to 16‐cell stage) explored expression heterogeneity between cells in each embryo. Cell expression profiles become increasingly diverse immediately following the first zygotic division, driven by both transcript partitioning error during mitosis and stochastic gene expression (Shi *et al*, 2015). Different groups of genes showed different behaviours, with some showing transiently or progressively increased variability. Few already variable genes become more variable after the 8‐cell stage: it is possible that transcriptional differences between cells in an embryo begin to become fixed at this time. Finally, the authors highlighted how the ratio of two genes’ expression may display particularly large amounts of heterogeneity due to asymmetric RNA distribution at mitosis, particularly if one or both of the initial transcripts is expressed at a low level. Given that many developmental decisions are specified by opposing lineage specifiers, stochastically driven heterogeneity in the expression of lineage specifiers seems a reasonable explanation for how symmetry can be broken.

Another study applied scRNA‐seq to mouse embryonic cells from the 2‐cell to 16‐cell stage of development (Goolam *et al*, 2016), identifying highly heterogeneous expression of *Sox2* and *Oct4* (master pluripotency regulators) gene targets at the 4‐cell stage. *Sox21* was identified as a gene of potential importance due to particularly heterogeneous expression across cells within an embryo and its joint regulation by *Sox2* and *Oct4*. Moreover, *Sox21* knockdown was shown to subtly bias cells towards an extraembryonic fate. Coupling the observed heterogeneity in *Sox21* expression with its fate‐biasing effect, it was suggested that this heterogeneity may be responsible for pushing cells towards specific lineages during early development. However, definitively identifying the origin of these heterogeneities remains a challenge.

**Figure 3:**
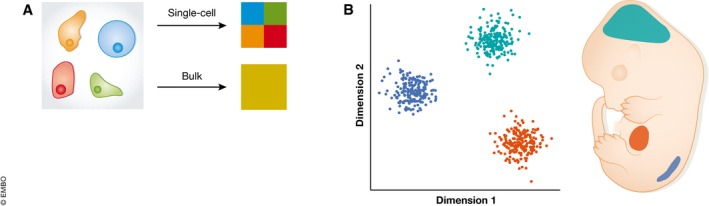
scRNA‐seq resolves cellular heterogeneity (A) While bulk gene expression assays provide an average read‐out of transcription over many cells, single‐cell RNA‐seq allows the assaying of gene expression in individual cells. (B) Single‐cell approaches facilitate working with complex systems such as embryos, where groups of cells with radically different expression profiles can be analysed without contamination from neighbouring tissues.

As development proceeds, cells become specialised into differentiated cell types through processes that are often summarised as a set of binary decisions. Single‐cell approaches are especially useful in this context, because they capture cells before, during and after lineage commitment, unlike the discrete population averages of bulk sequencing (Fig 3).

One study has analysed gastrulation in the mouse, capturing epiblast cells at embryonic day (E) 6.5 along with mesodermal cells (marked using the cell‐surface marker *Flk1*) at E7.0, E7.5 and E7.75 (Scialdone *et al*, 2016). Different cell types were readily identified, with pseudotime constructed over the blood precursor lineage recapitulating known gene expression changes and facilitating identification of new marker genes.

Using these data as an “atlas” of normal embryonic development allowed the authors to investigate how perturbations to developmental mechanisms affect cells’ expression patterns and the cell types that they can differentiate into. A common hypothesis, driven by work in embryonic stem cell systems, states that cell fate commitment follows a path of binary choices. In the mesodermal lineage analysed here, *Tal1* is a transcription factor essential for specification of the blood lineage through an unknown mechanism of action. Under a binary decision model, *Tal1* ^−/−^ cells would necessarily differentiate to a cardiac lineage in the absence of this key transcription factor, as supported by *in vitro* studies (Org *et al*, 2015).

The authors generated *Tal*1 knockout embryos, applied scRNA‐seq to the mesodermal lineage and computationally mapped cells from the *Tal1* ^−/−^ embryos on to the clusters identified from wild‐type cells. This allowed proper comparison between similar cell types between the two sets of embryos while controlling for compositional changes.

Cells from the mutant embryos did not map to the blood progenitor or erythroid clusters, consistent with the absence of *Tal1*. However, cardiac markers were not upregulated in the *Tal1* ^−/−^ cells, unlike observations *in vitro* (Van Handel *et al*, 2012). Because the cells were not committing towards the cardiac fate, the findings called into question whether binary cell fate choices previously reported from *Tal1* knockout cells are an *in vitro* artefact, or instead occur at a later stage *in vivo* (Van Handel *et al*, 2012).

### Developmental trajectories

A particular advantage of single‐cell methods is the ability to capture cells at various developmental stages in a single experiment. It is possible to reconstruct developmental pathways using the variety of cell states assayed using techniques motivated by the concept of pseudotime (see Fig 2 and section “State‐of‐the‐art analysis techniques”, above). Using this cell ordering, it is possible to inspect how cells change over the course of development, and which genes are critical for driving progression. This approach has been applied very widely and here we discuss some examples of how it has provided insight from different gene expression measurement technologies.

Cultured embryonic stem cells offer a versatile platform for following developmental pathways, as different morphogens can guide their development into a number of different tissues. One example is a study of the development of human definitive endoderm cells (Chu *et al*, 2016). In this study, cells were ordered along the developmental pathway, successfully reconstructing the behaviour of known markers. This ordering allowed the discovery of novel candidate regulators; for example, a driver of definitive endoderm differentiation (KLF8) was identified and validated by testing for changes in the fraction of differentiated cells post‐KLF8 knockdown.

Trajectory inference is not limited to transcriptome data. Single‐cell protein expression data (acquired by mass cytometry) have been used to identify the developmental progression of B cells in human bone marrow (Bendall *et al*, 2014). In addition to identifying a developmental progression consistent with known marker proteins, rapid changes in protein expression along pseudotime were used to identify points of cellular coordination—these correspond to the checkpoints that define progression between developmental stages. Additionally, changes in the structure of the regulatory network of STAT5 along B‐cell development were noted.

Mesodermal cells from 7‐ to 8‐day‐old mouse embryos were analysed using single‐cell qPCR to understand the early development of blood lineages (Moignard *et al*, 2015). Here, diffusion maps were used to identify developmental pseudotime trajectories (Haghverdi *et al*, 2015), recovering correctly the ordering of known markers. Cell states were defined via binarisation of the expression data, and a network was constructed that linked cells through changes in a single gene's expression state. This facilitated a mechanistic interpretation of the data, where predicted gene regulators were supported by motif searches and, for *Erg1*, validated in reporter systems.

Finally, it has also been shown that developmental trajectories inferred from chromatin‐accessibility assays correspond closely to those inferred from expression information (preprint: Pliner *et al*, 2017).

Coupling information from different expression modalities along developmental trajectories offers potential for improved experimental design. For example, rare but important cell types could be identified using very high‐throughput proteomic or flow cytometry techniques, before using identified markers to sort cells for transcriptome‐wide analysis with scRNA‐seq.

### Allele‐specific expression

Biases of expression of different alleles is a difficult problem to dissect in bulk populations: Is it driven by subpopulations of cells that express only one allele at a time, or by a consistent but small bias across all cells? How much does the noisy process of transcription affect the way individual alleles are expressed?

To assay allele‐specific expression (ASE) at the single‐cell level, experiments must be designed carefully. Library preparation should ideally follow a protocol that allows reads to be generated across the whole length of the transcript [e.g. Smart‐Seq2 (Picelli *et al*, 2014)], to maximise the number of inter‐allele polymorphisms that can be assayed. Additionally, a system with the greatest possible number of allelic sequence differences is preferred. A frequently used system is the F1 hybrid mouse, that is the offspring of two different inbred lines.

The first single‐cell RNA‐seq study of ASE used early‐stage mouse embryos (up to the blastocyst stage) and adult tissues (Deng *et al*, 2014), observing a high rate of monoallelic expression (12–25%) for even highly expressed autosomal genes. Cells in the same embryo expressed different genes monoallelically, implicating chance in deciding which alleles are expressed in individual cells. Similar behaviour has been observed in primary human fibroblasts (Borel *et al*, 2015), suggesting that stochastic monoallelic expression is common across many species and cell types.

While certain genes are known to produce predictable allele‐specific expression patterns (i.e. imprinted and sex‐biased genes), many genes display expression from a specific allele chosen apparently at random. This type of allele‐specific expression is referred to as autosomal random monoallelic expression (aRME). aRME describes a heritable attribute of gene expression, for which single‐cell analysis provides a particularly useful experimental tool. Reinius *et al* (2016) applied single‐cell RNA‐seq to clonal cell populations, showing that less than 1% of genes demonstrating aRME had conserved behaviour; this is in contrast to previous bulk RNA‐seq work that observed aRME for over 7% of assayed genes (Gimelbrant *et al*, 2007). This single‐cell work hints at the very dynamic nature of transcription (as expressed alleles change at least as fast as the cell cycle) and a lack of coordination between expression of different alleles.

**Figure 4:**
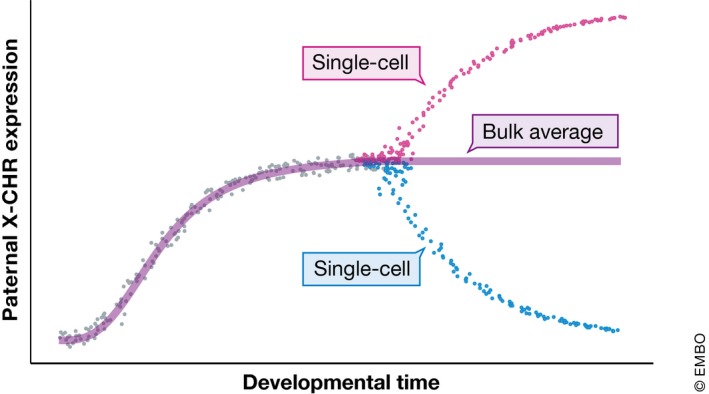
Allele‐specific expression at single‐cell resolution By exploiting single nucleotide polymorphisms in single‐cell RNA‐seq reads, it is possible to quantify how much individual alleles contribute to a gene's total expression. For developmental biology, this can be applied to study, for example, when monoallelic expression patterns become set during embryonic development and how they relate to fate decision, as in the case of X chromosome inactivation (Chen *et al*, 2016).

Allele‐specific expression is a useful tool for studying X chromosome inactivation (XCI), the process by which the dosage of X chromosome genes is controlled between sexes in mammals (Fig 4). Experiments in both mice (Chen *et al*, 2016) and humans (Petropoulos *et al*, 2016) showed that the process is asynchronous across cells and that gene expression from the silenced X chromosome is gradually and uniformly reduced. One interesting difference between the two is that *Xist* is biallelically expressed during XCI in humans and monoallelically expressed in mice.

### Lineage tracing

Nearly all measurements of gene expression kill the cell, providing a snapshot of cellular development but losing information about a cell's lineage. As a cell's lineage represents a history of the decisions that cells have made during development, it is closely intertwined with cell fate choice. Assays have now been developed to reconstruct cell lineage alongside the capturing of expression data.

The most direct approach for identifying lineage relationships between cells using sequencing technologies lies in the genome. The pattern of mutations that individual cells acquire over time is passed on to their daughter cells upon division—a lineage tree can therefore be constructed from the distributions of these mutations across cells. However, single‐cell whole‐genome sequencing is expensive and presents many technical challenges (Gawad *et al*, 2016).

**Figure 5:**
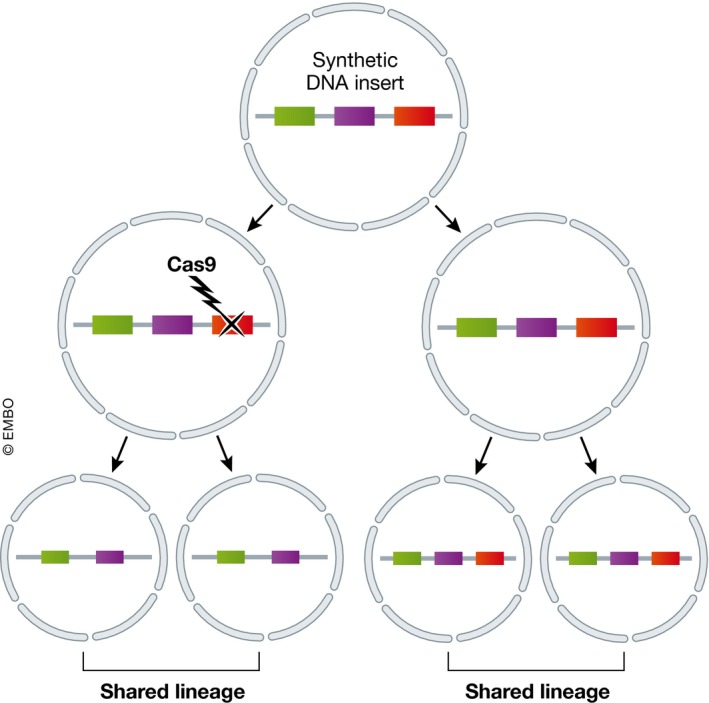
Lineage tracing Understanding how cells are related to each other is central to understanding how developmental processes work. However, comparison of transcriptomic profiles does not allow the reconstruction of these lineage relationships. Recent approaches used CRISPR/Cas9 to mutate a synthetic DNA construct, providing a genomic or transcriptional read‐out containing cell lineage information.

In particular, the relative infrequency of neutral mutations per cell cycle makes lineage determination over short timescales difficult. Given this, two techniques have been designed to implement CRISPR/Cas9 genome editing via a synthetic construct within a cell, which can accumulate mutations in a rapid manner. One of these methods provides output via imaging (Frieda *et al*, 2017) and the other via transcriptome or genome sequencing (McKenna *et al*, 2016). Both rely on the editing of a DNA‐inserted barcode: endogenously expressed Cas9 (with an appropriate guide RNA) progressively and randomly alters this barcode, leaving permanent sequence changes that are inherited by daughter cells. The cell may transcribe the barcode, amplifying its presence within the cell, from where the sequence can be read out by probe labelling (Frieda *et al*, 2017), by RNA‐seq (preprint: Raj *et al*, 2018) or simply by DNA sequencing (McKenna *et al*, 2016). The similarities and differences between cells’ barcodes catalogue the mutational history of the assayed cells, and therefore the lineage relationship between them (Fig 5).

The sequencing approach was applied to zebrafish embryos by McKenna *et al* (2016), showing that adult organs were derived from only a small number of progenitor cells and that individual ancestral progenitor cells contributed to multiple organs and germ layers. The imaging approach has been demonstrated by a proof‐of‐concept study in mouse embryonic stem cells (Frieda *et al*, 2017).

Such a scarring system may be made inducible by some signal provided experimentally or naturally within a biological system. This adaptation allows for improved lineage resolution at particularly important time points.

### Spatial transcriptomics

Cellular decision‐making is heavily influenced by a cell's environment and the signals it receives from its neighbours. However, existing scRNA‐seq techniques require tissue dissociation, thereby discarding spatial information. Recovering this information has been the subject of several computational investigations.

**Figure 6:**
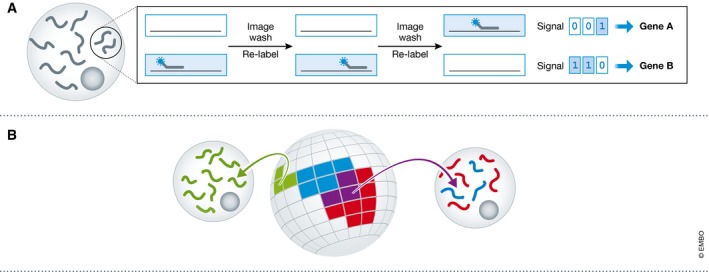
Spatial gene expression data (A) Most single‐cell gene expression assays require dissociation of tissues, destroying locational information. New *in situ* hybridisation methods, however, offer high‐throughput transcriptomic quantification captured alongside intra‐ and inter‐cellular localisation. (B) In the absence of such techniques, others have used reference “atlases” to map back sequenced cells onto structures with known expression patterns.

Several groups have utilised gene expression atlases onto which cellular expression profiles can be remapped (Fig 6B). One approach used existing *in situ* hybridisation maps of spatially restricted genes as a “barcode” to which the complete expression profiles of individual cells can be matched. This was applied by two groups to reconstruct expression patterns in zebrafish embryos (Satija *et al*, 2015), and to the brain of the marine annelid *Platynereis dumerilii* (Achim *et al*, 2015). This type of approach is particularly useful where the biological structure is robust between samples, or where many high‐quality reference data sets exist.

Where the system considered is known to have a robust or invariant structure, it is possible to reconstruct pseudospatial information from scRNA‐seq expression data alone. Scialdone *et al* (2016) used an unsupervised approach to position cells along the anterior–posterior axis of the primitive streak during gastrulation, identifying genes expressed posteriorly (biasing cells towards, e.g., blood fate) and those expressed anteriorly (biasing cells towards, e.g., endoderm). Despite successes with post hoc reconstruction, methods that preserve spatial information experimentally will likely prove more accurate and generalisable, particularly to tissues with complex structure. Consequently, several groups have worked to develop such techniques.

The recently developed methods of merFISH (Chen *et al*, 2015) and seqFISH (Shah *et al*, 2016b) use sequencing‐by‐hybridisation techniques for transcriptomic quantification. In these assays, fixed cells are subject to repeated washes of fluorescently labelled DNA probes coupled with matched rounds of imaging; careful design of the probes allows individual RNA species to be identified by different sequences of fluorescence across washes, building up a unique barcode for each transcript (Fig 6A). The accuracy and resolution of these techniques have been improved by sample background clearing (Moffitt *et al*, 2016; Shah *et al*, 2016a), but the number of genes that can be reliably assayed has remained much lower than can be achieved with scRNA‐seq (e.g. 249 genes in Shah *et al*, 2016b). However, recent efforts have reported the quantification of over 10,000 different transcripts in the same cells (Eng *et al*, 2017).

Locational information in these FISH assays is encoded at the individual transcript level, allowing the examination of intra‐cellular effects (e.g. organelle localisation) as well as inter‐cellular influences. These imaging techniques offer vast potential in developmental biology, particularly with regard to understanding signalling processes in complex systems such as embryos.

## The importance of perturbations in single‐cell analyses

High‐throughput ‐omics techniques have found their forte in hypothesis generation: because they quantify vast amounts of information, they offer considerable scope for identifying differences between samples that can form the basis of future targeted studies. However, in and of themselves, changes in gene expression levels do not provide conclusive evidence for hypotheses: Are cellular phenomena driving or being driven by the expression change? Is the expression change a function of some orthogonal effect? Have apparently significant changes arisen by chance? Follow‐up experiments are therefore critical—by inducing over‐ or underexpression of a gene, strong signals should be detectable from further ‐omic assays, or through cellular behaviour alone. An appealing alternative exists for single‐cell transcriptomics: natural variation in expression levels. As cells stochastically express more or less of individual genes than other cells in a population, differences in overall gene expression should propagate through gene regulatory networks, forming a large set of “micro‐perturbations”. However, such small differences can be readily confounded by technical artefacts (e.g. batch effects), and inference of gene regulatory networks from scRNA‐seq data has been challenging to date. For instance, the SCENIC package utilises cis‐regulatory information to reinforce transcriptional gene network learning (Aibar *et al*, 2017).

One possible solution to this problem is the combination of single‐cell RNA‐seq with targeted CRISPR screens to produce more impactful perturbations at high throughput (Adamson *et al*, 2016; Dixit *et al*, 2016; Jaitin *et al*, 2016; Datlinger *et al*, 2017). Implementations of this approach are Perturb‐seq and CROP‐seq. Specifically, these methods infect pools of cells with viral constructs containing guide RNAs, which together with endogenously expressed Cas9 protein can target specific areas of the genome. Single‐cell RNA‐seq can then be applied to profile the transcriptome of each cell in addition to the specific guide RNAs that were transduced, linking a holistic view of gene expression with the knowledge of which perturbations have caused these transcriptional changes. Because of the pooled nature of such experiments and the ability to tune the multiplicity of infection, it is possible to load a large assortment of guide RNAs into a single experiment, allowing the investigation of a complex set of interacting perturbations without needing to massively increase the experiment's scale.

## The future of single‐cell transcriptomics in developmental biology

Already single‐cell transcriptomics has had a transformative effect in developmental biology: the ability to assay individual cells has facilitated the study of highly heterogeneous but small cell populations from the earliest stages of development. Moving forward, there are several areas where new developments will lead to even deeper insights than have already been obtained.

Perhaps most obviously, the vast majority of single‐cell experiments performed to date divorce the spatial location of a cell from its transcriptional profile. Especially in early development, where spatial location affects the signals that a cell receives and thus its eventual fate, marrying these two sources of information will be extremely powerful. New approaches that increase the throughput of multiplexed RNA FISH, and other *in situ* sequencing technologies, promise to make this a reality. One important challenge will be to computationally record the location of individual cells within the embryo using a common coordinate framework—this will facilitate cross‐sample comparisons. Interestingly, such a framework has already begun to be developed within the context of the Allen Brain Atlas (Sunkin *et al*, 2013) and will be an important challenge for the nascent Human Cell Atlas project (Regev *et al*, 2017). Extending this to early development will be critical, with effective work in the fly having already begun (Karaiskos *et al*, 2017).

Once generated, these spatially resolved maps of expression within the embryo will facilitate computational inference of signalling gradients, enabling both known and novel morphogen patterns to be found. This will play a key role in understanding how cells incorporate signalling information to make decisions about their downstream fate. While interesting, such new hypotheses will have to be complemented by additional experiments, for example involving the use of conditional knockout models.

Another key area where technology is driving biological discovery is the ability to assay multiple molecular layers within the same cell. Recent advances have allowed the epigenome, transcriptome and chromatin accessibility of the same cell to be profiled (preprint: Clark *et al*, 2018), therefore allowing insight into the mechanisms driving changes in gene expression. When coupled with information about a cell's location in the embryo (and the associated signalling gradients introduced above), we will begin to move towards a holistic model of cell fate choice and, indeed, of embryogenesis itself.

Underpinning all of these advances will be developments in computational methods. It is critically important that computational methods are developed in parallel with new technologies and that computational biologists work in close partnership with the experimental laboratories generating the data. Together, the potential for transforming our understanding of development is tremendous.

## Conflict of interest

The authors declare that they have no conflict of interest.
